# Arthroscopic Radial Styloidectomy: An All‐Dorsal Two‐Portal Surgical Technique

**DOI:** 10.1002/atn2.70164

**Published:** 2026-07-01

**Authors:** Frank L. Vazquez, Anna L. Gorsky, Kier Blevins, Krishna N. Chopra, Nina Suh, Eric R. Wagner

**Affiliations:** ^1^ Department of Orthopaedic Surgery Emory University School of Medicine Atlanta Georgia U.S.A.

## Abstract

Radial‐sided wrist pain and impingement are recognized sources of persistent symptoms following 4‐corner fusion, often related to radioscaphoid contact and progressive degenerative changes. Radial styloidectomy has been described as a surgical option to address this pathology while preserving carpal stability. An arthroscopic approach offers the potential advantages of improved visualization, reduced soft tissue disruption, and concomitant treatment of intra‐articular pathology. Standard wrist arthroscopy portals are established, with the 6R portal for visualization and the 3‐4 portal as the working portal. Diagnostic arthroscopy allows for evaluation of the radiocarpal joint and treatment of concomitant pathology, including synovitis, which may be addressed with arthroscopic synovectomy. The 3‐4 portal is then used for visualization and the 6R portal for working on the remainder of the case. The radial styloidectomy is performed arthroscopically using a 3.0 oval burr and a 4.0 bone cutter. Fluoroscopy confirms a 4 mm resection of the radial styloid to relieve impingement while maintaining the extrinsic ligaments and wrist stability. Patients start therapy at 2 weeks, moving on to strengthening at 6 weeks. Arthroscopic radial styloidectomy is an effective surgical approach for wrist pain and impingement in post‐4‐corner fusion patients. This minimally invasive technique provides significant pain relief and facilitates early functional recovery.

## Graphical abstract

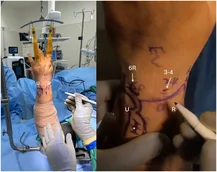

**VIDEO 1:** Annotated procedure video of arthroscopic radial styloidectomy. Video content can be viewed at https://doi.org/10.1002/atn2.70164.

Persistent wrist pain following 4‐corner fusion (4CF) can present a therapeutic challenge, particularly when associated with radioscaphoid impingement.^1^, ^2^ In post‐4CF patients, localized pain at the radial styloid may arise from altered load transmission and joint mechanics, often exacerbated by wrist radial deviation.^3^ Dynamic impingement between the radial styloid and trapezium is a recognized source of pain in these patients and can lead to radiocarpal arthritis.^4^, ^5^, ^6^

Nonoperative treatments such as nonsteroidal anti‐inflammatory drugs, splinting, and activity modification are typically first‐line management for post‐4CF wrist pain.^7^, ^8^, ^9^ However, in cases of persistent symptoms or mechanical impingement, surgical intervention may be warranted.^8^ Surgical options for symptomatic post‐4CF impingement include total wrist arthrodesis, wrist arthroplasty, neurectomy, and radial styloidectomy.^4^, ^8^ Although total wrist fusion offers definitive pain relief, it sacrifices motion.^10^ In contrast, radial styloidectomy preserves motion and can be performed via an open or arthroscopic approach.^11^ The arthroscopic technique, in particular, allows targeted resection, preservation of soft tissue stabilizers, and potentially faster recovery with lower morbidity.^11^ Indications include radiotrapezial impingement with focal radiocarpal arthritic changes, especially in patients without significant pancarpal degeneration.^11^, ^12^ Contraindications include pancarpal osteoarthritis or concern for ligament attenuation or wrist instability that may be further exacerbated by resection of the radial styloid and its ligamentous attachments.^13^, ^14^

The traditional technique for a radial styloidectomy involves an open dorsal approach, which puts the extrinsic ligaments of the wrist at risk for iatrogenic damage.^15^ Alternatively, traditionally, the arthroscopic radial styloidectomy involved the use of 3 portals including the standard 3‐4, but then the slightly more dangerous 1‐2 and volar radial portals.^11^ Alternatively, we describe a technique for arthroscopic radial styloidectomy using two standard wrist arthroscopy portals, including the 3‐4 and 6R. This technique allows for safe portal creation, while paying particular attention to preserving extrinsic ligamentous integrity and optimizing visualization for safe and effective resection. We often combine this technique with a selective neurectomy.^16^

## SURGICAL TECHNIQUE

Arthroscopic radial styloidectomy is indicated for patients with persistent radial‐sided wrist pain and mechanical impingement following 4CF who have failed nonoperative management and do not show pancarpal degenerative changes or wrist instability. The procedure is designed to relieve radiocarpal impingement while preserving extrinsic ligamentous integrity and carpal stability. The following describes a standardized, all‐dorsal arthroscopic technique utilizing two conventional wrist portals, which allows for safe and controlled resection under direct visualization and may be performed alone or in combination with selective wrist denervation.

### Surgical Setup

**FIGURE 1:**
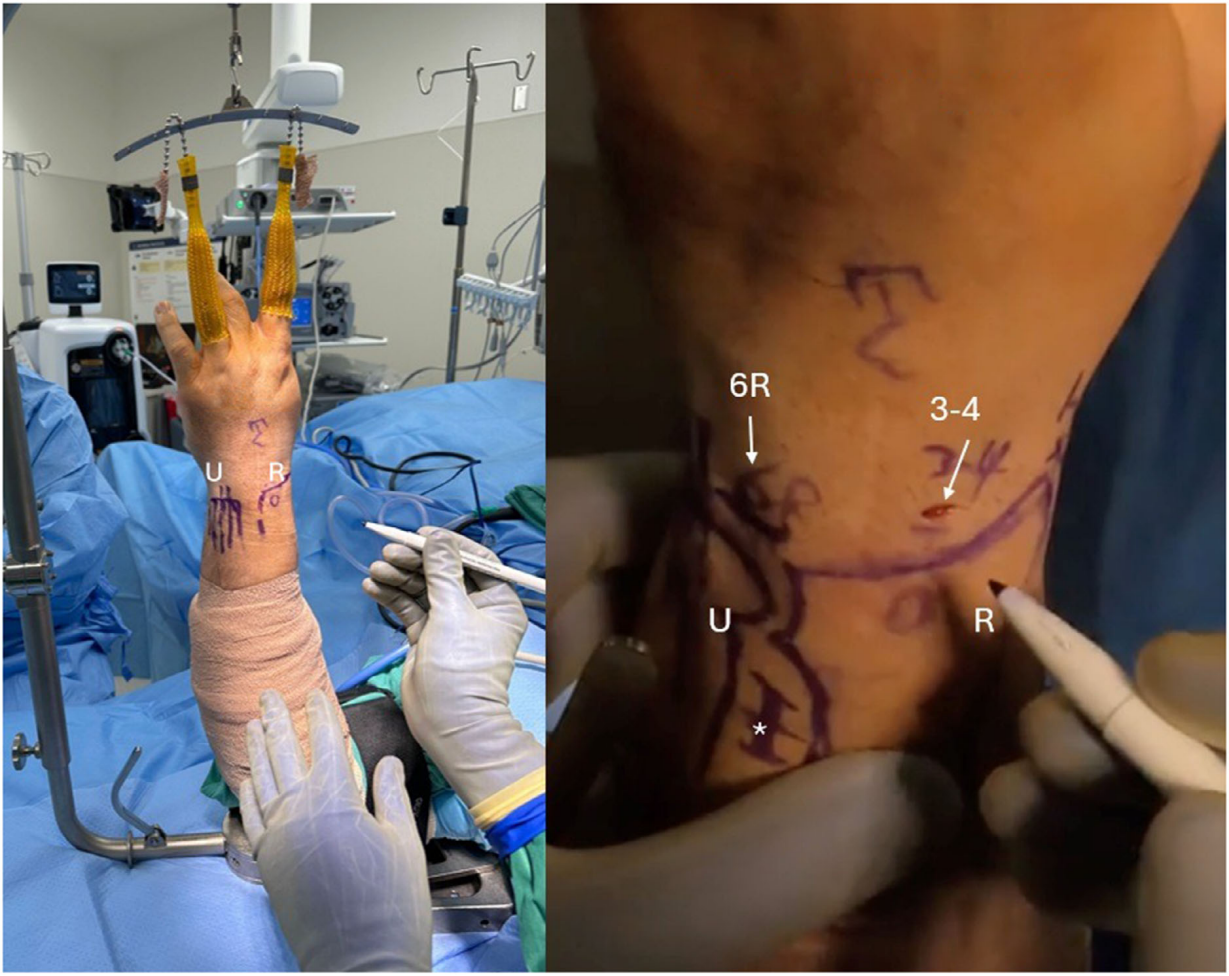
Left, supine on hand table in wrist traction tower. The operative wrist (left) is set up in a standard wrist arthroscopy tower and traction. The 3‐4 and 6R portals are marked, and landmarks are drawn out, including the ulna, radius, and DRUJ. A 1 cm dorsal incision will be made 2 cm proximal to the DRUJ (*). The ulnar side is labeled with a U, and the radial side is labeled with an R. (DRUJ, distal radioulnar joint.)

The patient is placed in the supine position on a hand table under general anesthesia, with appropriate padding of bony prominences and sequential compression devices applied for deep vein thrombosis prophylaxis. The operative limb is set up on a standard wrist arthroscopy tower and traction (Figure 1). It is then prepped and draped in a sterile fashion, and a standard surgical timeout is performed. A regional anesthetic block is administered using a mixture of 1% lidocaine with epinephrine. A pneumatic tourniquet is then inflated. The wrist is then distracted to 25 to 30 lbs. of traction.

### Arthroscopic Radial Styloidectomy

**FIGURE 2:**
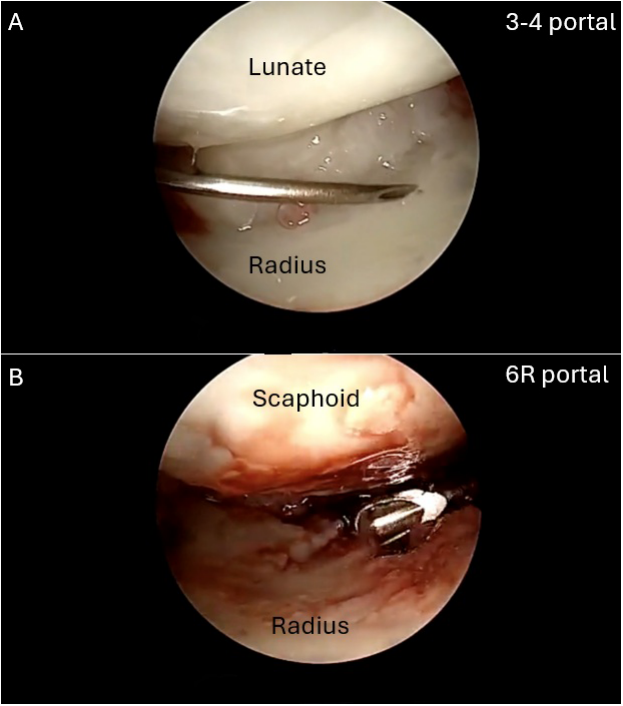
Left, supine on hand table in wrist traction tower. (A) Viewing from the 3‐4 portal, the 6R portal is established under direct visualization. (B) The 6R portal is utilized as the viewing portal, and the 3‐4 portal becomes the working portal, with a shaver inserted.

**FIGURE 3:**
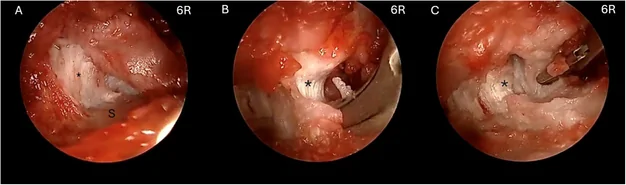
Left, supine on hand table in wrist traction tower. (A) Viewing from the 6R portal, there is fibrous and scarred capsule (*) adjacent and along the S. (B,C) Debridement of fibrous tissue is performed both dorsally and volarly using a biter. (S, styloid.)

A 3‐4 portal is established, and under direct visualization from the 3‐4 portal, the 6R portal is established (Figure 2A). A standard diagnostic arthroscopy and debridement is performed. Then, the viewing is switched to the 6R portal and using the 3‐4 as the working portal (Figure 2B). After bluntly opening the capsule, diagnostic wrist arthroscopy is performed. If synovitis is visualized, an extensive synovectomy may be performed dorsally and volarly (Figure 3A‐C).

**FIGURE 4:**
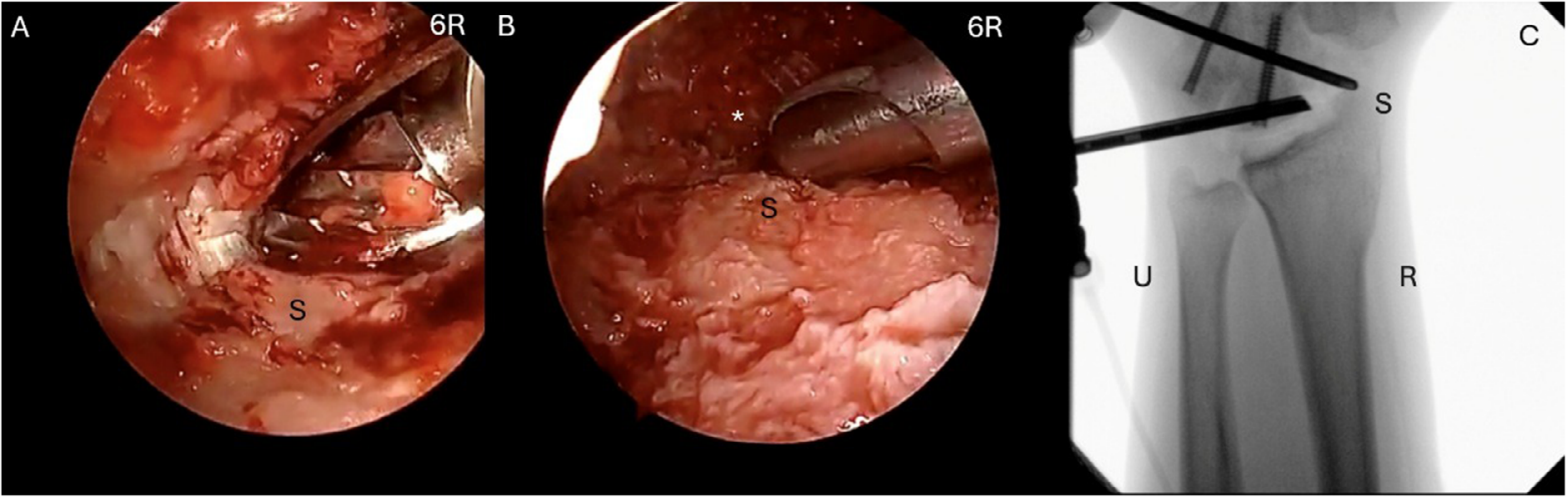
Left, supine on hand table in wrist traction tower. (A) The radial styloidectomy is begun with a 3.0 oval burr and resected up to the midpoint where the S meets the scaphoid fossa under fluoroscopic guidance. (B) A 4.0 hooded bone cutter is used to complete the resection around the edges of the S in order to preserve the critical extrinsic wrist ligaments and stabilizers (*). (C) Approximately 4 mm of radial S bone is removed under direct visualization and fluoroscopic guidance. (R, radial; S, styloid; U, ulnar.)

**FIGURE 5:**
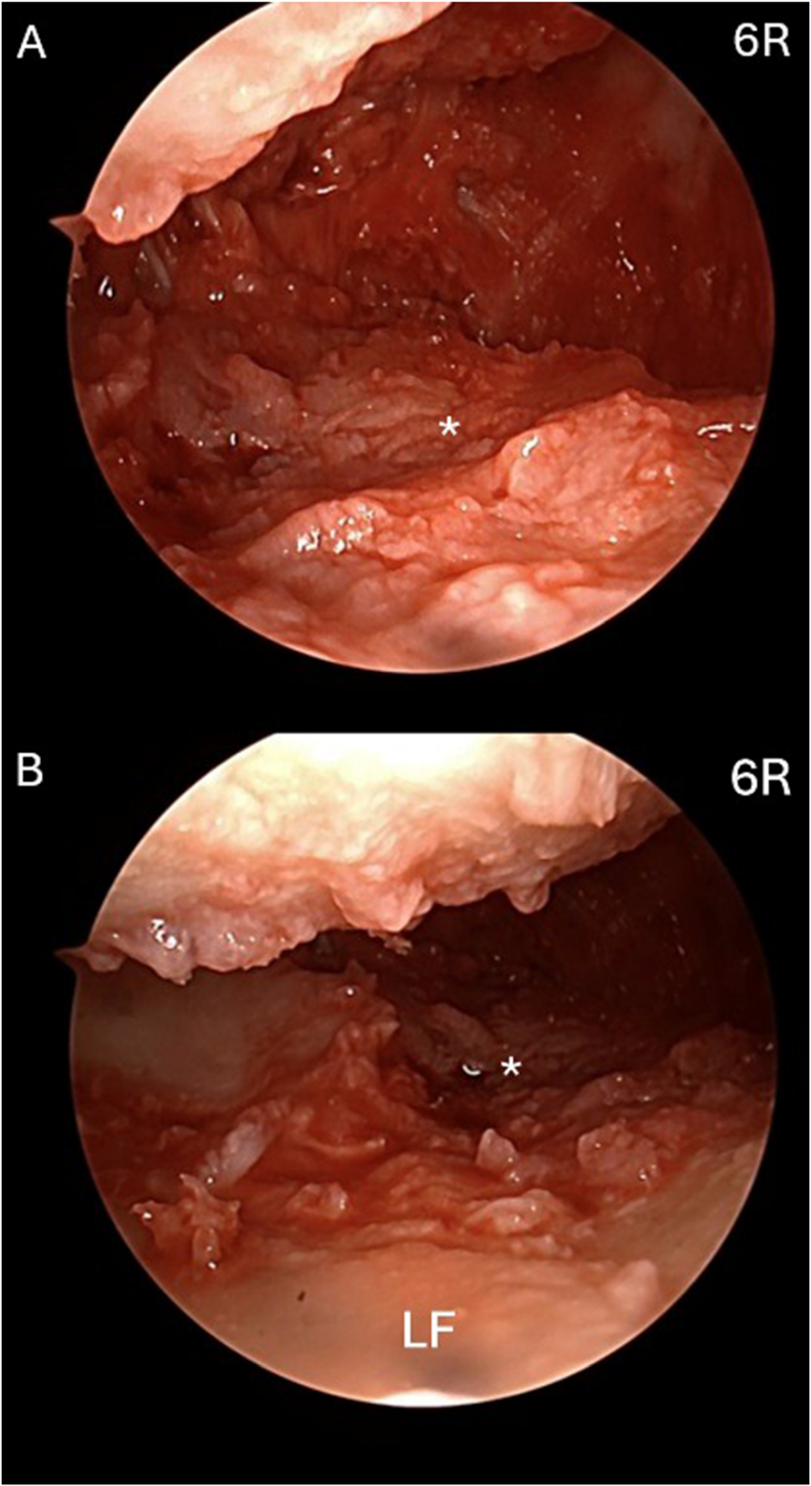
Left, supine on hand table in wrist traction tower. (A) Viewing from the 6R portal, the final styloidectomy resection (*) is visualized with intact capsule and ligaments. (B) Viewing from the 6R portal, the LF and remnant fossa (*) with adjacent resection bed. (LF, lunate fossa.)

For the radial styloidectomy using the 6R as a viewing portal and 3‐4 as the working portal, a 3.0 oval burr (Arthrex, Naples, FL) is used to start the styloidectomy. This is used to take down the central part of the styloid, resection up to the point where it meets the midpoint of the scaphoid fossa. To get the edges while protecting the critical extrinsic ligaments, a 4.0 bone cutter (Arthrex, Naples, FL) is used to finish the resection, pushing the hood against the ligaments around the edges of the styloid. Ultimately, the goal is to resect approximately 4 mm of the styloid, with the assistance of fluoroscopic guidance, ensuring preservation of ligamentous and ligamentous integrity (Figures 4A‐C and 5A,B). Final arthroscopic and fluoroscopic images are obtained to confirm adequate resection (Video 1).

### Posterior Interosseous Nerve and Anterior Interosseous Nerve Neurectomies

A 3 cm longitudinal dorsal incision is made approximately 2 cm proximal to the distal radioulnar joint. Dissection proceeds sharply under loupe magnification through the skin and subcutaneous tissues, exposing the extensor tendons. The posterior interosseous nerve and posterior interosseous artery are identified on the interosseous membrane. The posterior interosseous nerve is neurolyzed, and a 2 cm segment is excised from proximal to distal. The interosseous membrane is split longitudinally, revealing the pronator quadratus and anterior interosseous nerve. The anterior interosseous nerve is similarly neurolyzed, and a 2 cm segment is excised.

After thorough irrigation, all incisions are closed with absorbable sutures. A compressive dressing and wrist splint are applied.

### Postoperative Protocol

**FIGURE 6:**
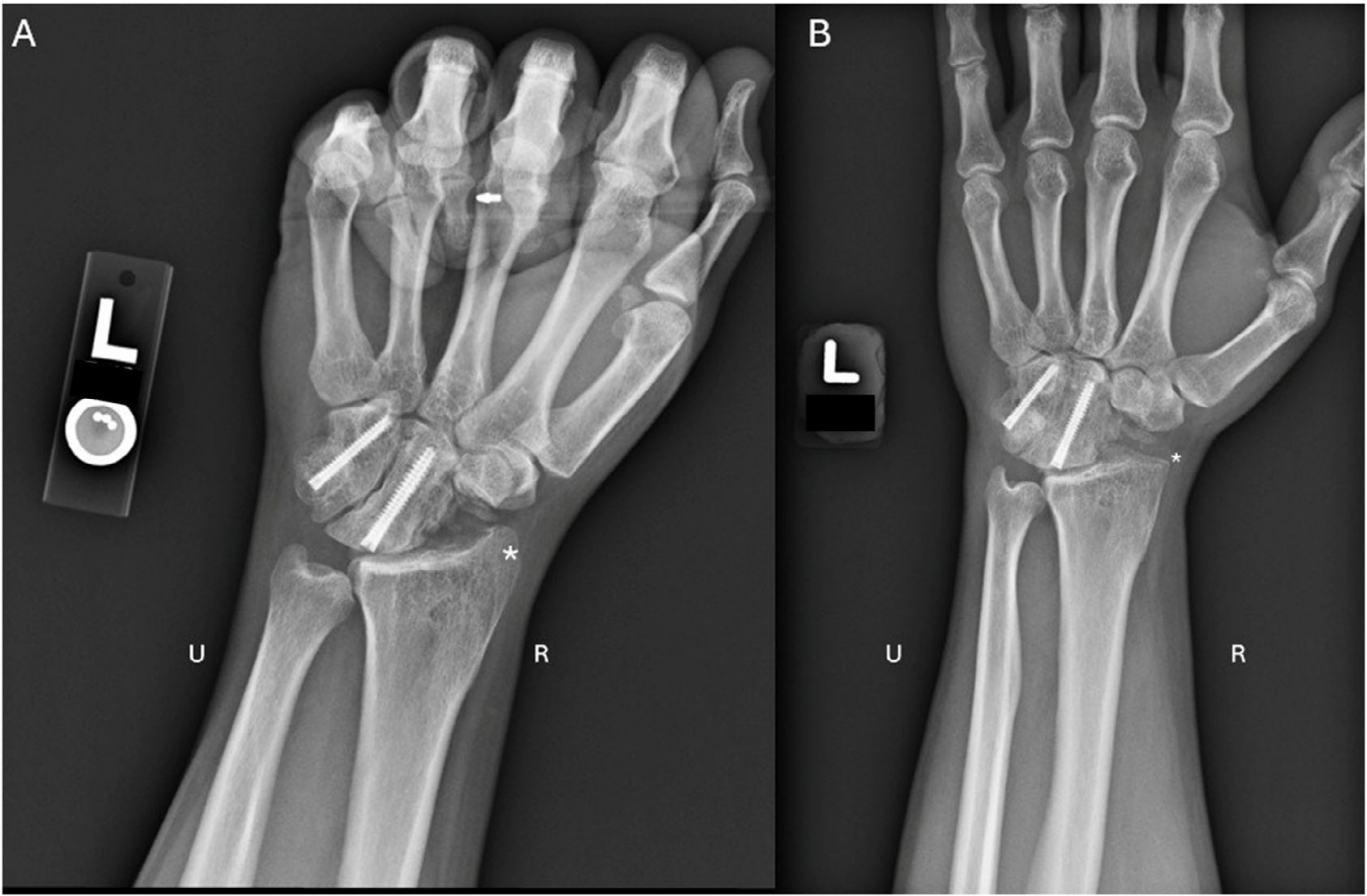
Left, supine on hand table in wrist traction tower. (A) Preoperative radiograph with prominent radial styloid (*). (B) 8‐week postoperative radiograph status post radial styloid resection (*). (R, radial; U, ulnar.)

**FIGURE 7:**
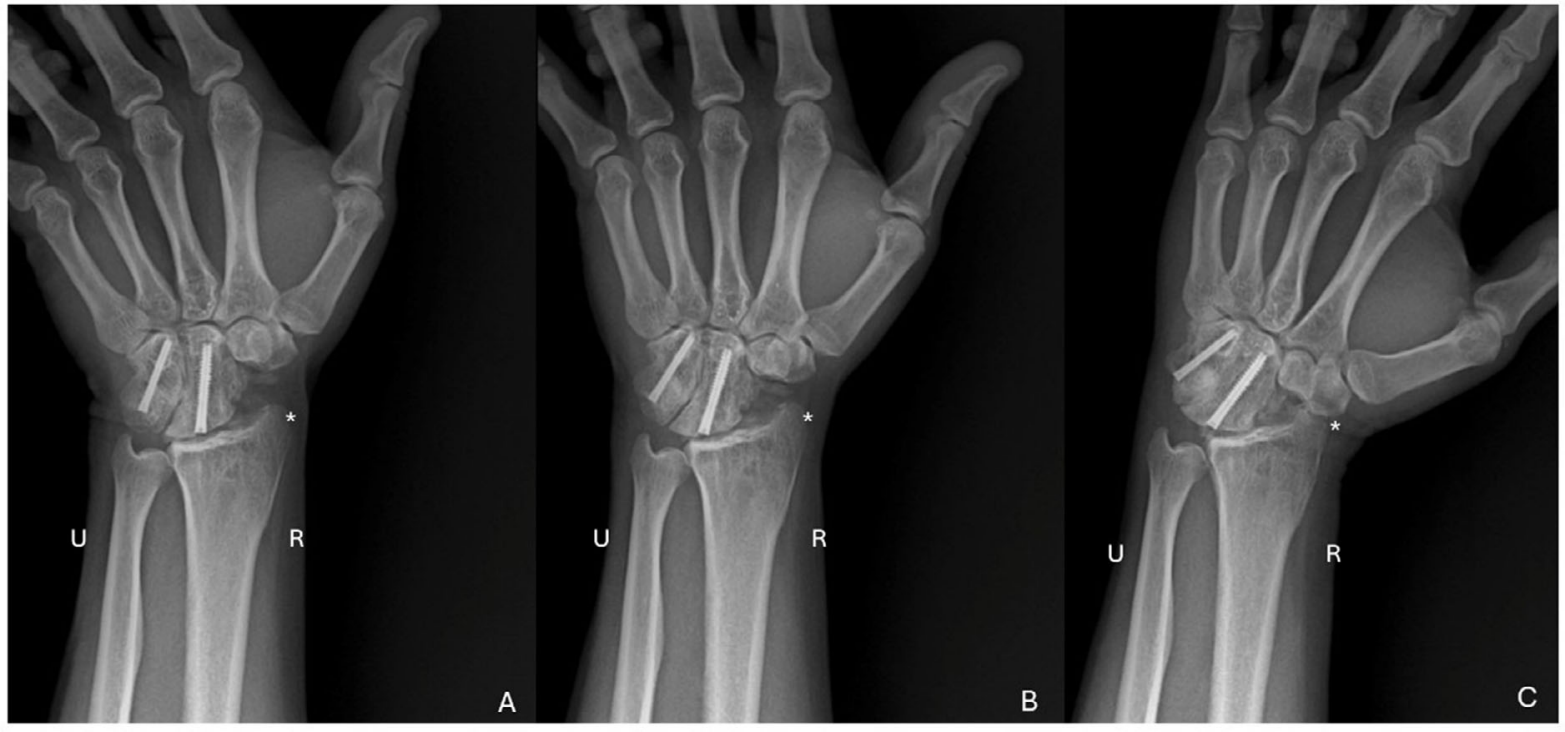
Left, supine on hand table in wrist traction tower. Preoperative dynamic wrist radiographs showing prominent radial styloid (*) impingement in (A) ulnar deviation, (B) neutral, and (C) radial deviation. (R, radial; U, ulnar.)

**FIGURE 8:**
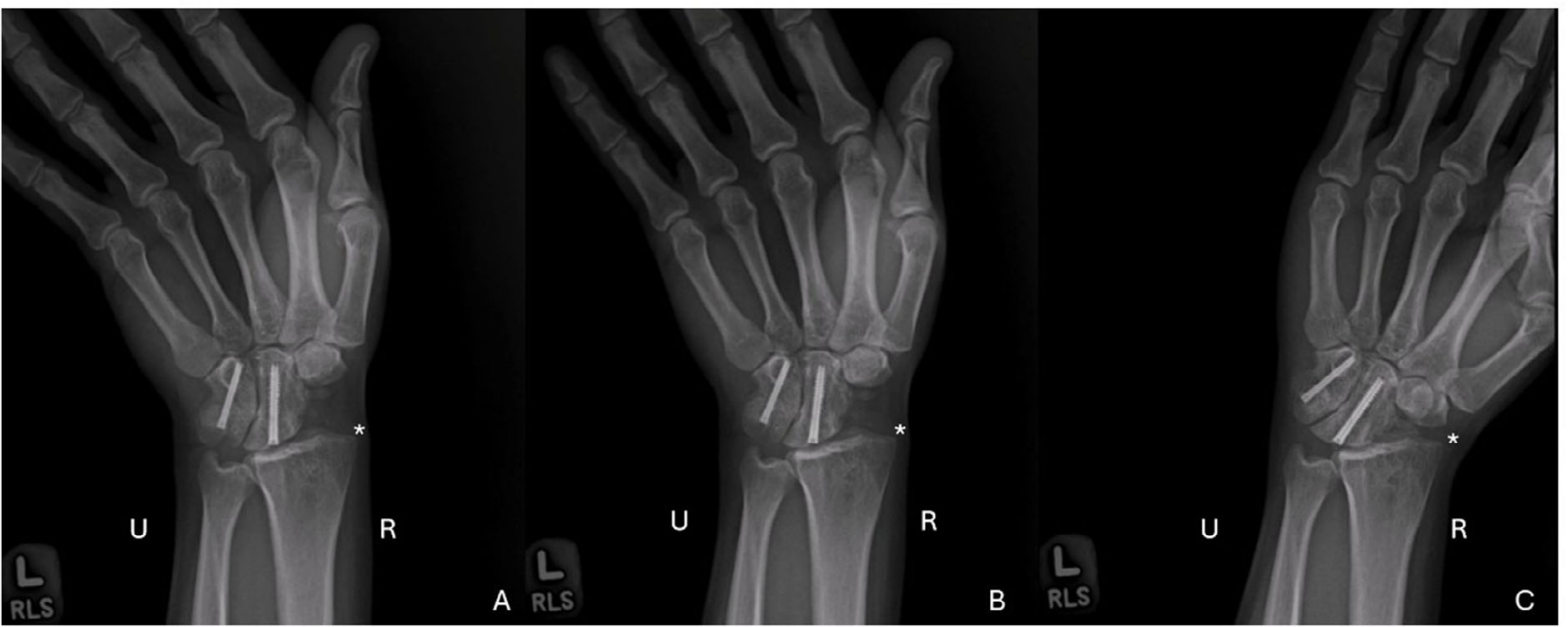
Left, supine on hand table in wrist traction tower. Eight‐week postoperative digital dynamic radiographs showing resected radial styloid (*) and impingement‐free range of motion in (A) ulnar deviation, (B) neutral, and (C) radial deviation. (R, radial; U, ulnar.)

Postoperatively, patients are placed in a volar‐based splint for 1 week. At the first postoperative visit, the dressing and splint are removed, and patients are allowed to wean from a removable splint while initiating a home exercise program focusing on the range of motion of the hand, wrist, and fingers. At the 8‐week follow‐up, radiographs are used to evaluate healing and ensure the absence of complications (Figure 6A,B). Digital dynamic radiograph may be used to evaluate for impingement through the ulnar and radial deviation range of motion (Figures 7A‐C and 8A‐C). The patient may return to all activities.

## DISCUSSION

We describe a safe, reproducible arthroscopic technique for radial styloidectomy with concomitant anterior and posterior interosseous neurectomy in patients experiencing persistent wrist pain and impingement following 4CF. This minimally invasive approach allows for targeted bony resection under excellent visualization and, combined with a selective denervation,^16^ helps to target many areas of pain within the wrist while preserving wrist motion and stability. Pearls and pitfalls of this technique are outlined in Table 1.

**TABLE 1:** Pearls and Pitfalls

Pearls	Pitfalls
Measure resection depth using both fluoroscopy and arthroscopy, using the burr size as a guide for 3‐4 mm	Excessive resection (>5 mm) may cause wrist instability and ulnar translocation from damage to extrinsic ligaments
Hooded instruments (i.e., bone cutter) allow for aggressive synovectomy and peripheral bone resection while protecting critical wrist extrinsic ligaments	Under‐resection at the styloid may lead to residual impingement
Adequate traction (25‐30 lbs.) is important for adequate visualization using the 6R portal	A thorough synovectomy should be performed to start prior to bony resection to be sure to see all aspects of the radial styloid
Localize end of styloid utilizing fluoroscopy intraoperatively before starting resection	

Radial styloidectomy is a recognized treatment for radiocarpal impingement in patients with focal arthritic changes or altered biomechanics postfusion.^11^, ^17^ Open techniques provide direct access but require more extensive soft tissue dissection and carry an increased risk of injury to surrounding nerves and ligamentous structures.^12^ In contrast, arthroscopic styloidectomy preserves soft tissue integrity and offers enhanced visualization of both the radial styloid and adjacent structures, including the volar ligaments, although it is technically demanding and requires precise portal management.^11^, ^12^ Given the proximity of the radial styloid to the superficial branch of the radial nerve, open procedures may carry a higher risk of iatrogenic nerve injury compared with arthroscopic techniques, which utilize smaller incisions and better visualization.^11^, ^18^ The improved visualization through arthroscopy also allows for accurate measurement of resection of the radial styloid at 3 to 4 mm, which reduces the likelihood of damaging critical stabilizing extrinsic ligaments. Excessive resection has been correlated with injury to adjacent ligamentous structures and, in severe cases, may result in complications such as ulnar translocation of the carpus.^12^, ^19^

Importantly, an anterior and posterior interosseous neurectomy may be performed as part of the same procedure. Selective wrist denervation is increasingly recognized as an effective adjunct in patients with chronic, localized wrist pain that is refractory to conservative measures.^16^, ^20^, ^21^, ^22^ Denervation targets nociceptive input while preserving motor function and proprioception.^21^ This allows for substantial pain relief without compromising wrist biomechanics or function.^21^, ^23^ Denervation of the anterior interosseous nerve and posterior interosseous nerve territories can significantly reduce pain perception from the central aspect of the wrist.^16^, ^24^, ^25^, ^26^, ^27^ However, a limitation is that it does not cover radial‐sided pain,^16^, ^27^ thus making it an ideal concomitant partner with the radial styloidectomy.^23^, ^26^, ^28^ By integrating both interventions, we leveraged the benefits of targeted anatomical correction with neuromodulatory control of pain, representing a comprehensive strategy for symptom relief.

In our technique, a standard dorsal wrist arthroscopy setup was used for the arthroscopic styloidectomy, which is in contrast to prior approaches. One of the original descriptions of an arthroscopic radial styloidectomy utilized the 3‐4 portal, combined with the slightly riskier 1‐2 and volar radial portals.^11^ The 1‐2 portal is in close proximity to the SBRN and radial artery, whereas the volar radial portal is also close to the radial artery, median nerve branches, and volar flexor tendons. Alternatively, we utilize the 6R as the primary viewing portal and the 3‐4 as the primary working portal. These are standard and safe portals without risks to neurovascular structures. And by using the 6R portal to see the entire articular surface of the distal radius, it enables the need for only 1 additional working portal. This reduces operative complexity, limits soft tissue dissection, and avoids additional risks associated with volar or radial portal placement near critical structures.^29^ It is important to maintain 25 to 30 lbs. of traction to enable excellent visualization. By utilizing the 6R and 3‐4 portals in a flexible manner, we maintained excellent visualization of the radial styloid, reduced soft tissue trauma, and minimized the risk of iatrogenic injury.

Despite the advantages of an all‐dorsal, 2‐portal arthroscopic approach, several risks and limitations warrant consideration. Arthroscopic radial styloidectomy is technically demanding and requires familiarity with wrist arthroscopy, precise portal placement, and careful fluoroscopic confirmation of resection depth. Inadequate visualization or insufficient synovectomy may result in under‐resection and persistent impingement, whereas excessive resection (>4‐5 mm) risks injury to critical extrinsic stabilizing ligaments, potentially leading to carpal instability^12^, ^30^ (Table 2).

**TABLE 2:** Advantages and Disadvantages

Advantages	Disadvantages
Minimally invasive approach with limited soft‐tissue disruption	Technically demanding; requires advanced wrist arthroscopy experience
Uses 2 standard dorsal portals (3‐4 and 6R), avoiding volar or radial portals near neurovascular structures	Visualization of the radial styloid is dependent on adequate wrist traction
Preserves extrinsic wrist ligaments through controlled, measured resection	Risk of instability if excessive bone resection is performed
Allows precise bony resection under combined arthroscopic and fluoroscopic guidance	Limited applicability in patients with pancarpal arthritis or ligamentous insufficiency
Can be combined with selective wrist denervation to address multifactorial pain	Concomitant denervation may confound isolated assessment of bony decompression

In conclusion, we describe an arthroscopic radial styloidectomy using a 2‐portal all‐dorsal technique that offer an efficient and safer alternative to 3‐portal techniques without compromising visualization or resection accuracy. When performed in combination with a selective neurectomy, it enables a multimodal approach to wrist pain associated with impingement and arthritis.

## DISCLOSURES

The author (E.R.W.) declares the following financial interests/personal relationships which may be considered as potential competing interests: E.R.W. receives consulting fees from Stryker, Smith and Nephew, DePuy Synthes, and Acumed; institutional research support from Konica Minolta; hospitality fees from Arthrex, Wright, Stryker, Integra, and Acumed. The other authors (F.L.V., A.L.G., K.B., K.N.C., N.S.) declare that they have no known competing financial interests or personal relationships that could have appeared to influence the work reported in this article.
